# Nanolipoprotein-Mediated Her2 Protein Transfection Induces Malignant Transformation in Human Breast Acinar Cultures

**DOI:** 10.1021/acsomega.1c03086

**Published:** 2021-10-26

**Authors:** Wei He, Angela C. Evans, William F. Hynes, Matthew A. Coleman, Claire Robertson

**Affiliations:** † Physical and Life Sciences Division, Lawrence Livermore National Laboratory, 7000 East Avenue, Livermore, California 94550, United States; ‡ Radiation Oncology, University of California Davis School of Medicine, 4501 X Street, Sacramento, California 95817, United States; § Materials Engineering Division, Lawrence Livermore National Laboratory, 7000 East Avenue, Livermore, California 94550, United States

## Abstract

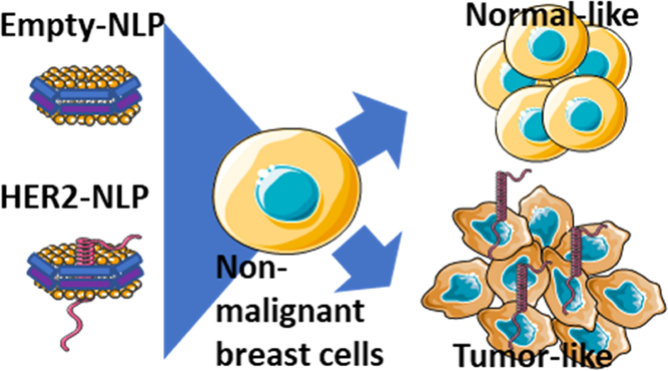

Her2 overexpression is associated with an aggressive form of breast cancer and malignant transformation. We demonstrate in this work that nanolipoprotein particles (NLPs) synthesized in a cell-free manner can be used to transfer Her2 protein into the membrane of nonmalignant cells in 3D culture in a nontoxic and facile manner. With NLP-mediated Her2 protein delivery, we observed an increased probability of nonmalignant cells forming apolar nongrowth-arrested tumor-like structures. The NLP delivery system alone or Her2-NLPs plus the Her2 inhibitor trastuzumab showed no effect on the acinar organization rate, indicating that Her2 signaling is key to this process. Transcriptomics revealed essentially no effect of empty NLPs compared to untreated cells, whereas Her2-NLPs versus either untreated or empty-NLP-treated cells revealed upregulation of several factors associated with breast cancer. Pathway analysis also suggested that known nodes downstream of Her2 were activated in response to Her2-NLP treatment. This demonstrates that Her2 protein delivery with NLPs is sufficient for the malignant transformation of nonmalignant cells. Thus, this system offers a new model for studying cell surface receptor signaling without genomic modification or transformation techniques.

## 1 Introduction

Nanolipoprotein particles (NLPs), which were originally developed as a mechanism to ensure correct folding of membrane-bound proteins, may represent a new mechanism of protein delivery to intact cells. These particles, comprised of lipid surrounded by apolipoproteins, allow for efficient solubilization of membrane-embedded proteins such as cell surface receptors,^1−6^ and the biomimetic lipid bilayer supports correct folding of membrane-embedded proteins^7,8^ when proteins are either harvested from cell or tissue cultures or expressed in cell-free lysates. Importantly, receptors such as ErbB2/Her2-supported NLPs retain tyrosine kinase functionality and sensitivity to Her2 inhibitors, even when synthesized in prokaryotic lysates in a one-pot manner.^5^

Recently, it was demonstrated that NLPs can be used to deliver a G protein-coupled receptor to cells without transgenic modification or artificial cell selection.^2^ In comparison to existing protein delivery systems that risk denaturing the proteins they carry, either by harsh packaging or endosomal lytic processes,^9−11^ NLPs maintain correct folding of mammalian cell surface receptors.^3,4,6^ NLPs, in contrast to other protein delivery systems, do not require cell membrane disruption^10,12−14^ and diffuse readily due to their small size relative to silica or poly(lactic-*co*-glycolic acid) (PLGA) nanoparticles,^2,5,15−17^ potentially resulting in a more efficient transfer of proteins into cells. Furthermore, NLP-mediated protein delivery is believed to be a spontaneous exchange of proteins between NLPs and plasma membrane, thus minimizing the potential off-target effects of the delivery system.^18^

Given the facile synthesis of NLPs and the simplicity of this protein transfection system, we sought to determine if this system could be used to rapidly engineer new oncoprotein-driven cancers. Half of all drugs that have been approved to treat cancer in the past decade target cell surface receptors.^19^ Despite the clinical success of these compounds, drug resistance remains an issue. For example, Her2/ErbB2/neu is a tyrosine kinase, which is overexpressed in 20% of breast cancers along with some gastrointestinal and other cancer types. Her2 (human epidermal growth factor family receptor 2, aka ERBB2) overexpressing breast cancers are typically treated with Her2 inhibitors such as trastuzumab, pertuzumab, and nivolumab. However, ∼15% of Her2-overexpressing cancers do not respond to these drugs at baseline,^20^ and most patients develop resistance within 1 year.^21^ Thus, understanding the biology of Her2 and its downstream signaling remains an urgent goal in cancer research.

Her2 is known to induce malignant transformation and activate PI3K and ERK pathway signaling, resulting in greater aggressiveness and likelihood of metastasis. In experiments where maintaining a diverse population of cells is necessary, this growth advantage provided by Her2 is an issue, as the highest expressors of Her2 will be selected for, even in the absence of applied selection pressure.^22,23^ Furthermore, recent work has demonstrated that transient activation of Her2 followed by suppression causes an inflammatory gene program, highlighting the need for temporal control of Her2 levels or activation state within cells.^24^ Some groups avoid this selection using artificially dimerizing Her2 transgene systems^25−27^ or drug-inducible Her2 constructs, but these systems can behave differently from wild-type Her2 and require genomic breaks and activating drugs.

Thus, we sought to determine whether Her2-NLPs could transform breast cells to take on malignant behavior and whether this culture system could be used as a new model of Her2-overexpressing breast cancer. Specifically, we determined the effects of Her2-NLPs in the breast acinar morphogenesis model, where nonmalignant cells cultured in three-dimensional (3D) laminin-rich gels form growth-arrested acinar-like structures that resemble normal breast, whereas malignant cells form apolar structures growing tumor-like colonies.^28,29^ Importantly, artificially dimerizing Her2 has been shown to induce malignant transformation and block acinar morphogenesis in similar models:^25−27,30^ if Her2 transported in NLPs is sufficient for malignant transformation, cells will organize into tumor-like structures instead of acinar-like structures. We found that NLPs readily transported Her2 to nonmalignant breast cells and induced tumor-like phenotypic changes and transcriptomic changes characteristic of Her2. These findings suggest that NLP transport of membrane proteins is sufficient to drive a healthy cell toward a complex disease phenotype without any further genetic or biochemical modification and avoids the various complications typically associated with such approaches.

## 2 Results

### 2.1 Her2-NLPs Transfer Functional Her2 Protein to Cells in 3D Culture

Her2-NLPs and control empty NLPs (lipid and apolipoprotein only) were synthesized in an *Escherichia coli* bacterial cell-free lysate and affinity-purified as previously described.^5^ We checked for the presence of endotoxin and found that empty NLPs contained an average endotoxin level of 104 EU/mg total protein, and Her2-NLPs contained 160 EU/mg total protein. When diluted to 5 μg/mL for cell culture, final endotoxin concentrations were less than 1 EU/mL, which represents a commonly used threshold for “endotoxin-free” cell culture media. Nonmalignant HMT3522-S1 cells cultured in 3D laminin-rich ECM hydrogels (as described previously^29^) were then stimulated with 5 μg/mL Her2-NLP or empty NLPs. Nonmalignant cells treated with Her2-NLPs demonstrated positive staining for Her2 at 18 h of culture (Supporting Information Figure S1).

### 2.2 Her2-NLPs Cause Nonmalignant S1 Cells to Disorganize into Malignant-like Structures

To determine the phenotypic consequences of NLP-mediated Her2 transfer, we cultured HMT3522-S1 and -T4-2 cells in 3D laminin-rich ECM (lrECM) hydrogels, as previously described.^29^ Briefly, single cells were dispersed in LrECM and allowed to gel, and then, they were overlaid with either culture media, or culture media + 5 μg/mL of Her2-NLPs. Media and NLPs were replaced every 2–3 days for 10 days. Samples were then fixed and stained to manually identify features associated with cellular polarity. Power analysis revealed that for a chi-square test assuming an effect size of 0.33 and a significance level of 0.05, scoring 100 structures would give a power of >0.8; thus, a total of 100 structures per condition per experiment were scored.

**Figure 1:**
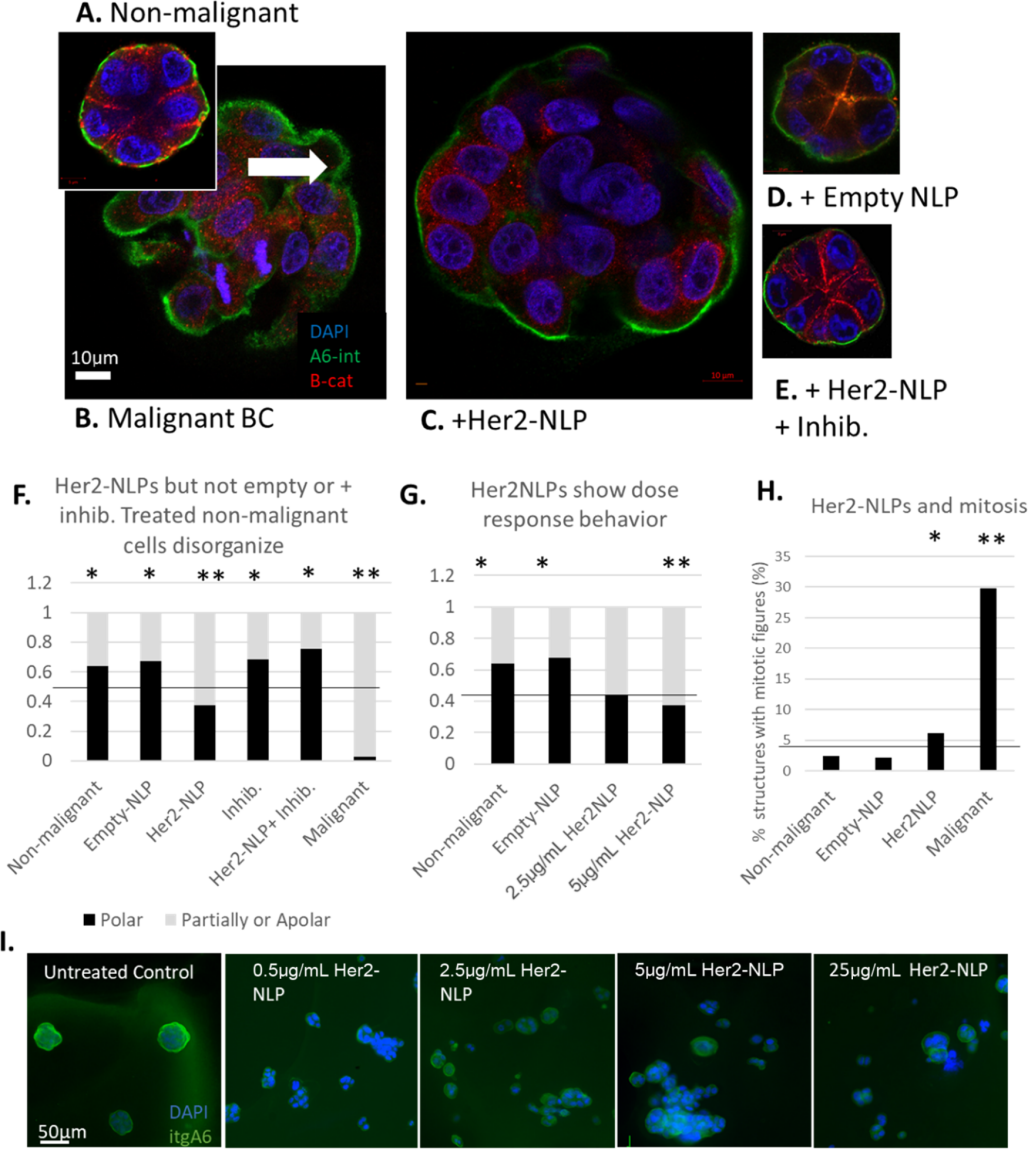
Her2 carried by NLPs causes malignant-like organization in nonmalignant cells. (A) Nonmalignant cells cultured in lrECM form growth arrested polarized acinar-like structure with the basal organization of α6 integrin (green) and lateral organization of β-catenin (red). (B) Malignant breast cancer cells form disorganized structures with no cell polarity, which fail to growth arrest, as shown by the presence of mitotic figures (white arrow). (C) Nonmalignant cells treated with Her2-NLPs form apolar masses, which fail to growth arrest. (D) In contrast, nonmalignant cells treated with empty NLPs organize normally. (E) Nonmalignant cells treated with Her2-NLPs and a Her2 dimerization inhibitory antibody organize normally. (F) Significantly more structures organize well in nonmalignant, empty-NLP-treated, inhibitory antibody only, and Her2-NLP+ inhibitory antibody conditions, whereas Her2-NLP-treated and malignant cells are less likely to organize into polarized, growth-arrested acini (*n* = 5 biological replicates, * indicates post hoc test significance of *p* < 0.05, ** indicates post hoc test of *p* < 0.001). (G) Her2-NLPs show dose–response behavior, with fewer structures organizing well with increasing NLP dosage (*n* = 3 biological replicates, * indicates post hoc test significance of *p* < 0.05, ** indicates post hoc test of *p* < 0.001). (H) Her2-NLP-treated structures are more likely to contain a mitotic figure than untreated or empty-NLP-treated cells (*n* = 3 biological replicates, * indicates post hoc test significance of *p* < 0.05, ** indicates post hoc test of *p* < 0.001). (I) Increasing the doses of Her2-NLPs from 0.5 to 25 μg/mL causes increasing abnormalities in the acinar structure.

As previously reported, the majority of nonmalignant S1 cells (Figure 1A) formed growth-arrested (<4% contained a mitotic figure) polar structures (60% ± well organized), whereas almost all malignant T4 (Figure 1B) cells formed apolar structures, with 30% of structures displaying 1 or more mitotic figures at the end of the culture. Her2-NLP-treated S1 cells (Figure 1C) were significantly more likely to disorganize and to contain mitotic figures compared to untreated S1 across five independent experiments (Pearson’s Chi-square test *p* < 10^–14^). To ensure that the observed effects were due to Her2 and not the nanocarrier system, we repeated NLP stimulation experiments with empty NLPs (Figure 1D) composed of lipid and apolipoprotein alone at 5 μg/mL, and with Her2-NLPs + a Her2 function-blocking antibody inhibitor (4C5-8, a trastuzumab biosimilar) at a 1:2 molar ratio (Figure 1E). Trastuzumab is a monoclonal antibody that is clinically used to treat Her2 positive breast cancer by binding to a conformational epitope of Her2 protein in its extracellular juxtamembrane domain and blocking its function. In our previous study, we have demonstrated that 4C5-8 binds to cell-free produced Her2-NLP similar to native Her2 protein,^5^ and in the current experiment, we observed no effect of either empty NLPs or Her2-NLPs in combination with Her2-targeted antibody that blocks the receptor function (Figure 1F). Titrating the Her2-NLP concentration revealed dose–response behavior, with increasing probability of disorganization with increasing levels of the delivered Her2 (Figure 1G–I). Increased numbers of cells containing mitotic figures were also observed in Her2-NLP-treated structures relative to either nonmalignant or empty-NLP-treated cells (Figure 1H).

To the best of our knowledge, this is the first demonstration in which the transfer of an oncoprotein is sufficient to drive the malignant transformation of cells in 3D culture. Specifically, we demonstrate that the Her2 receptor transferred to nonmalignant breast cells induces malignant-like growth patterns in a subset of cells and that this effect is not seen either with the protein transfer system (NLP alone) or Her2-NLP in concert with a conformational dimerization inhibitor. Akin to previous work using artificially dimerizing Her2,^26^ our work demonstrates that Her2 can block acinar morphogenesis; however, we use wild-type Her2 protein instead of relatively complex transgenic strategies. This work is distinct from previous experiments that studied the effects of Her2 using genomic modification, as we do not induce genomic breaks, or induce an immune response to cytoplasmic DNA^31^^31^ or select cells. We did not directly measure the dimerization state in these models; however, treatment with a Her2 dimerization inhibitor blocked the effect of Her2-NLPs, indicating correct folding and presentation of the receptor post-transfer to the breast cells.

### 2.3 Her2-NLPs Cause Transcriptomic Changes in Nonmalignant Cells

**Figure 2:**
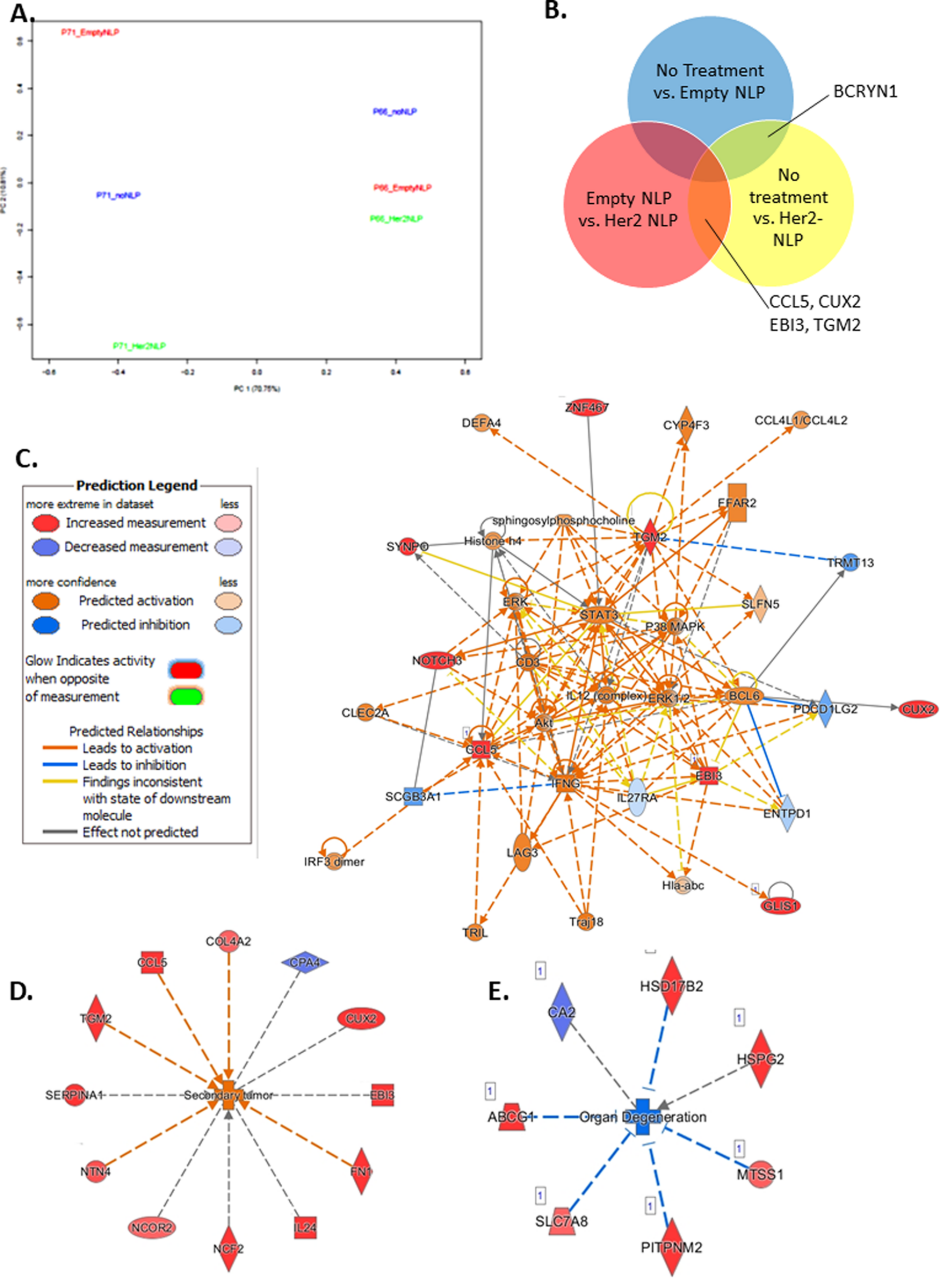
Her2-NLPs induce gene expression changes in nonmalignant 3D cultures. (A) PCA plot for all sample sequences shows that principal component 1 separates biological replicates, and principal component 2 separates Her2-NLP treated from other conditions. (B) Genes common to multiple comparisons include BCRYN1 for all NLP-treated cells versus untreated samples; and CCL5, CUX2, EBI3, and TGM2 for Her2-NLP-treated samples versus either empty-NLP or untreated samples. (C) Ingenuity pathway analysis (IPA) network comparing Her2-NLPs to empty-NLP disks predicts relationships among several biological families and transcriptional regulators involved in cancer progression and metastasis, including (D) ERK, Akt, p38MAPK, and STAT3. Genes in red indicate hits found in the dataset. Orange coloring indicates predicted activation of biomolecules, and blue indicates predicted inhibition. Deeper color saturation indicates more confidence in predicted regulation. (E) Secondary tumor formation is the top activated disease network associated with Her2-NLP treatment compared to untreated samples. (F) Organ degeneration is predicted to be inhibited with Her2-NLP treatment compared to untreated samples. Genes in red indicate upregulated genes found in the dataset, whereas genes in purple indicate those that were downregulated in the dataset. Orange coloring indicates predicted activation, and blue indicates predicted inhibition of the biological phenotype. Log_2_ fold change ±, *p* < 0.05.

To understand the transcriptomic changes induced by increased levels of Her2, we cultured nonmalignant S1 cells with no treatment, treated with 5 μg/mL empty NLP, or treated with 5 μg/mL Her2-NLP in 3D LrECM for 8–10 days. Cells were then extracted from the lrECM hydrogel and then lysed to extract total RNA for two biologically independent experiments. The RNA was isolated and enriched through poly-A selection and sequenced (HiSeq, Illumina). Reads were normalized, then mapped to the human genome and compared across groups using DESeq2 using a *p*-value cutoff of <0.05 and an absolute log_2_ fold change of >1. Principal component analysis (PCA) revealed that replicate was the primary cause of variability between experiments, followed by Her2-NLP treatment (Figure 2A).

Comparing untreated and empty-NLP-treated cells revealed only one differentially expressed transcript, the noncoding lncRNA BCYRN1. Gene ontology analysis also revealed no differentially expressed terms, further emphasizing the minimal effects of the NLP system (Figure 2B and Supporting Information Figure S2). In contrast, comparing empty NLPs and Her2-NLPs revealed nine upregulated genes, and comparing no treatment and Her2-NLPs revealed 32 upregulated genes and six downregulated genes. Four genes were consistently upregulated with Her2-NLP treatment as compared to either empty NLP alone or untreated cells (Figure 2B). Transcripts identified in Her2-NLP-treated groups relative to either untreated or empty-NLP-treated factors overlapped with previously reported screens for acinar morphogenesis (EBI3, CCL5)^32^ and for residual disease in Her2+ breast cancer (CCL5^33,34^), breast cancer outcome (EBI3^35−37^), and metastasis (TGM2,^38^ EBI3^37,39^).

Differentially expressed genes comparing Her2-NLP treatment to empty NLPs or to untreated cell cultures were analyzed via ingenuity pathway analysis (IPA). Her2-NLP-treated cells compared to either untreated or empty-NLP-treated cells showed a clear activation of cancer-related biological responses (Supporting Information Table S2) and predicted activation of signaling nodes, which have been linked to Her2 overexpression such as Stat3,^40^ ERK and Tgfβ,^30^ p38MAPK,^41^ and NFκB^32^ (Figure 2C and Supporting Information Figure S3). Notably, signatures of cancer-related malignancies, including breast and ovarian cancers, were identified as prominently associated with Her2-NLP treatment as compared to empty NLPs (Supporting Information Figure S4). The strongest disease networks associated with Her2-NLP treatment were activation of secondary tumor formation and suppression of organ degeneration (Figure 2D,E). Her2-NLP treatment was also linked with several cancer-related disease behaviors, including proliferation, the synthesis of reactive oxygen species, and the formation of cellular protrusions. Upstream factor analysis suggested that common upstream nodes may include NFκB, lipopolysaccharide, interferon γ, and tumor necrosis factor (TNF) (Supporting Information Table S3). The predicted activation of breast cancer-associated signaling nodes suggests that our model recapitulates features of Her2 overexpressing breast cancer and may offer new avenues to study Her2 signaling in cells that are difficult to transfect or in cell populations, where maintenance of genome heterogeneity is key.

## 3 Conclusions

Our demonstration that Her2-NLPs induce malignant-like phenotypic and transcriptomic changes shows that this model represents a discrete oncoprotein-driven model of malignant transformation. This protein delivery system is currently limited to membrane-bound proteins, but given the centrality of the surface receptors in cancer, including the tyrosine kinase receptor family^42^ and the G protein-coupled receptor family,^43^ there is a clear potential for widespread use of this system in rapidly engineering receptor-driven cancers, which represent targets for over half of all cancer drugs approved in the last decade. Future work using this cellular modification system can take advantage of the lack of alternative splicing of Her2, dynamic introduction of Her2 for pulse-chase experiments, and use the reversible nature of this system to study Her2 withdrawal.

## 4 Experimental Section

### 4.1 NLP Preparation

Her2-NLPs were prepared as previously described.^5^ Briefly, plasmids encoding for human full-length ErbB2 gene and a truncated 6x-His-tagged apolipoprotein A1 (Δ49A1) were synthesized using the cell-free Expressway system (Life Technologies) in the presence of 1,2-dimyristoyl-*sn*-glycero-3-phosphocholine (DMPC, Avanti). After overnight expression, Her2-NLPs were harvested from the cell-free mixture by native nickel pulldown.^5^ The purified NLP stocks then underwent buffer exchange into pH 7.4 PBS and sterile filtration for use in antibiotic-free mammalian cell cultures. Empty NLPs were made using the same cell-free method except that ErbB2 plasmid was absent. Protein concentrations were determined using NanoDrop. Endotoxin concentrations were determined using the Endosafe-PTS^TM^ (Charles River) endotoxin testing system based on the Limulus amebocyte lysate assay and are expressed as EU/mg total protein.

### 4.2 Cell Culture

Human immortalized breast cells (S1) and breast cancer cells (T4-2) from the HMT3522 progression series (kind gift of Mina Bissell)^44^ were maintained in DMEM/F12 (11330, Thermo Fisher) supplemented with 250 ng/mL insulin (I6634, Sigma-Aldrich), 10 μg/mL apo-transferrin (T2252, Sigma-Aldrich), 2.6 ng/mL sodium selenite (Corning, 47743-618), 10^–10^ M β-estradiol (E2785, Sigma-Aldrich), 1.4×10-6 M hydrocortisone (H0888, Sigma-Aldrich), 5 μg/mL ovine prolactin (LA Biomedical Research Institute), and for S1 only, 10 ng/mL EGF (11376454001, Roche). S1 were seeded in uncoated T75 flasks (Corning) at 2e^4^/cm^2^, re-fed every 2 days, and passed every 7 days, and T4-2 were seeded in collagen-coated flasks (Corning) at 1e^4^/cm^2^, re-fed every 2 days, and passed every 5 and 3 days, respectively. Cells were kept in humidified incubators at 37 °C and 5% CO_2_ supplementation, and CO_2_ calibrations were performed biweekly with a test kit (Fyrite, Bacharach). Cells were screened for mycoplasma contamination every 2 months (MycoAlert, Lonza).

### 4.3 Acinar Morphogenesis Assay

S1 or T4-2 cells were passaged, counted, and resuspended in 100% lrECM (354230, growth factor reduced Matrigel, Corning) at 800k cells/mL or 600 k/mL, respectively on ice. Cells in lrECM were transferred to dishes, allowed to gel for 20 min at 37 °C, then covered with complete culture media ± 5 μg/mL Her2-NLPs or empty NLPs or Her2-NLPs plus a Her2 blocking antibody (clone 4D5, MCA6092, Biorad) at 26.67 μg/mL (which represented 1:1 molar ratio with Her2-NLPs). Media was replaced every 2–3 days for 10 days.

At day 10, cultures were harvested for imaging by removing the culture media and smearing the cell-laden lrECM onto coated glass slides (Superfrost Plus, Thermo). Slides were then immediately fixed in 10% formalin for 15 min and washed 3× in PBS, blocked in for 1 h at room temperature in IF buffer composed of 3% bovine serum albumin (BSA) and 0.5% Triton X-100 in PBS supplemented with 10% normal goat serum and goat anti-mouse Fab fragment (115-007-003, Jackson ImmunoResearch) to block mouse antibodies present in the lrECM. Primary antibodies for β-catenin (Ab32572 Abcam), α-6 integrin (555734, BD Biosciences) and laminin (L9393, MilliporeSigma) were diluted 1:100 in IF buffer and stained for 2 h at room temperature. Slides were then washed 3× in IF buffer, and secondary antibodies (A21429, A-11006, A32723 as appropriate; Thermo Fisher) were applied at 1:500 dilution in IF buffer for 1 h at room temperature, followed by 3 washes with IF buffer and 3 washes with PBS. Slides were then stained with DAPI at 0.1 μg/mL for 5 min and mounted (ProLong Gold, Thermo Fisher) and coverslipped.

Slides were imaged on a laser scanning confocal microscope (LSM700, Zeiss) equipped with an Acro plan 40x/1.1NA water immersion lens. Then, 100 structures per experiment were scored by a trained observer according to criteria in Supporting Information Table S1. Pearson’s Chi-square test and Bonferroni post hoc tests were performed in R.

### 4.4 Transcriptomics

After 10 days of culture, cells were harvested from lrECM by incubation with 5mM EDTA in PBS on ice for 30 min with gentle agitation and spun down to collect structures. Cell pellets were then snap-frozen on dry ice for shipping, followed by RNA extraction, poly-A capture, cDNA synthesis, end repair, and adapter ligation. Samples were then sequenced (HiSeq, Illumina). Reads were trimmed to remove adapter sequences and poor-quality nucleotides and then aligned to the Homo sapiens reference genome (GRCh38). Differentially expressed genes were compared using DEGseq. 2. The Wald test was used to generate *p*-values and log_2_ fold changes. Genes with an unadjusted *p*-value < 0.05 and absolute log_2_ fold change >1 were called differentially expressed genes for each comparison. A gene ontology analysis was performed on the statistically significant set of genes by implementing the software GeneSCF v.1.1-p2. The goa_human GO list was used to cluster the set of genes based on their biological processes and determined their statistical significance. To estimate the expression levels of alternatively spliced transcripts, the splice variant hit counts were extracted from the RNA-seq reads mapped to the genome. Differentially spliced genes were identified for groups with more than one sample by testing for significant differences in read counts on exons (and junctions) of the genes using DEXSeq. The data discussed in this publication have been deposited in NCBI’s Gene Expression Omnibus (Edgar et al., 2002)^45^ and are accessible through GEO Series accession number GSE184648 (https://www.ncbi.nlm.nih.gov/geo/query/acc.cgi?&acc=GSE184648).

### 4.5 Ingenuity Pathway Analysis (IPA)

Differentially expressed genes from RNA-seq studies were run through IPA (Qiagen). In total, three core analyses were run: empty-NLP vs no treatment, Her2-NLP vs no treatment, and Her2-NLP vs empty-NLP. Both direct and indirect relationships were considered for IPA mapping and statistical analysis. Log_2_ fold change cutoffs of ±1 and *p*-values < 0.05 were included in the analysis.

## Supporting Information Available

Demonstration of Her2 uptake by S1 cells, gene expression changes with Her2-NLP or empty-NLP treatment compared to untreated cells, ingenuity pathway analysis for Her2-NLP treatment, gene expression changes associated with cancers, tables of acinar scoring criteria, association of diseases and biofunctions activated or inhibited by Her2-NLP treatment, and upstream factor analysis for Her2 treatment (PDF)
