# Supplementary material for: Nanolipoprotein-Mediated Her2 Protein Transfection Induces Malignant Transformation in Human Breast Acinar Cultures
Source: ACS Omega. 2021 Oct 26;6(44):29416–23. doi: 10.1021/acsomega.1c03086 (PMC8581977; doi:10.1021/acsomega.1c03086)
Supplement: Supplementary file 1 — ao1c03086_si_001.pdf [file ao1c03086_si_001.pdf]

Supplementary Files: Nanolipoprotein mediated Her2 protein transfection induces malignant transformation in human breast acinar cultures

Wei He[2], Angela C. Evans[3], William F. Hynes[1], Matthew A. Coleman[2,3], Claire Robertson[1*]

1: Materials Engineering Division, Lawrence Livermore National Lab. 7000 East Ave, Livermore, CA, 94550, USA

2: Physical and Life Sciences Division, Lawrence Livermore National Lab. 7000 East Ave, Livermore, CA, 94550, USA

3: Department of Radiation Oncology, University of California Davis, School of Medicine, Sacramento, CA, 95817, USA

* Correspondence to Claire Robertson, Robertson40@llnl.gov

Figure S1: Her2-NLPs are taken up by cells. A&B: An untreated cell cultured in 3d lrECM does not show any staining for Her2, whereas C&D: a treated cell shows abundant Her2 staining throughout the membrane and cytoplasm, but not nucleus.

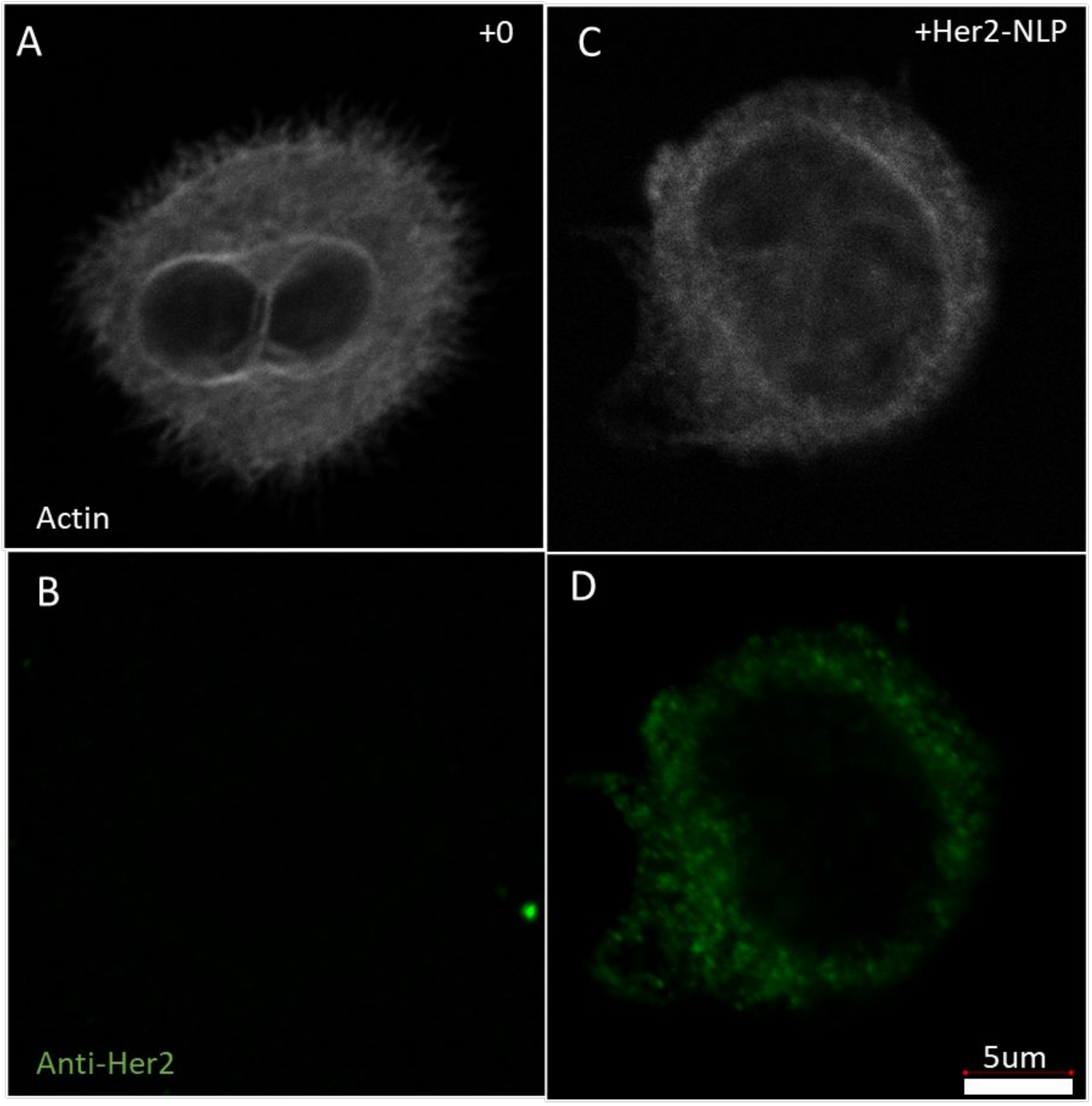

Figure S2: Gene expression changes associated empty or Her2-NLP treatment. A: Table of differentially expressed genes across all comparisons shows 1 DEG for no-treatment vs. empty NLP treatment. B: List of differentially expressed genes for all comparisons studied. Genes highlighted in yellow are common to both Her2-NLP treated comparisons and in green are common to either NLP treated condition.

A.

Comparison	Up	Down	Total DEG
No_treatment-vs-empty_NLP	0	1	1
empty_NLP-vs-Her2_NLP	9	0	9
No_treatment-vs-Her2_NLP	32	6	38

B.

No Treatment v. Empty
BCYRN1
**Empty v. Her2-NLP**
NOTCH3
EBI3
CUX2
SYNPO
GLIS1
ZNF467
TGM2
AC009133.2
CCL5

No Treatment v. Her2-NLP
IL32
CYP24A1
NTN4
HSD17B2
PITPNM2
SLC7A8
SEMA6A
CA2
EBI3
CUX2
FN1
KYNU
NCF2
ADM2
CPA4
COL4A2
HSPG2
RNF175
ABCG1
SLC2A6
IL24
C15orf39
RNF150
MTSS1
SPSB4
SAMD11
NCOR2
SERPINA1
TGM2
RBM20
LBH
LINC00863
BCYRN1
SHANK3
AL132780.2
IGFL2-AS1
CCL5
AL161431.1

Figure S3: Her2-NLP treatment activates the regulation of several key biological pathways when compared to untreated cells. Networks identified via Ingenuity Pathways Analysis (IPA) show connections between differentially expressed genes in our dataset and cancer progression and immune regulated biomarkers, such as A: RAS and TNF, B: ERK, TGFB and PI3K, and C: ERK, p38MAPk, and NFkB. Genes in red indicate up-regulated genes found in dataset, whereas genes in purple indicate those that were down-regulated in the dataset. Molecules in gray indicate associated connections as predicted through IPA. Log_2 fold change cutoff +/-1, p<0.05.

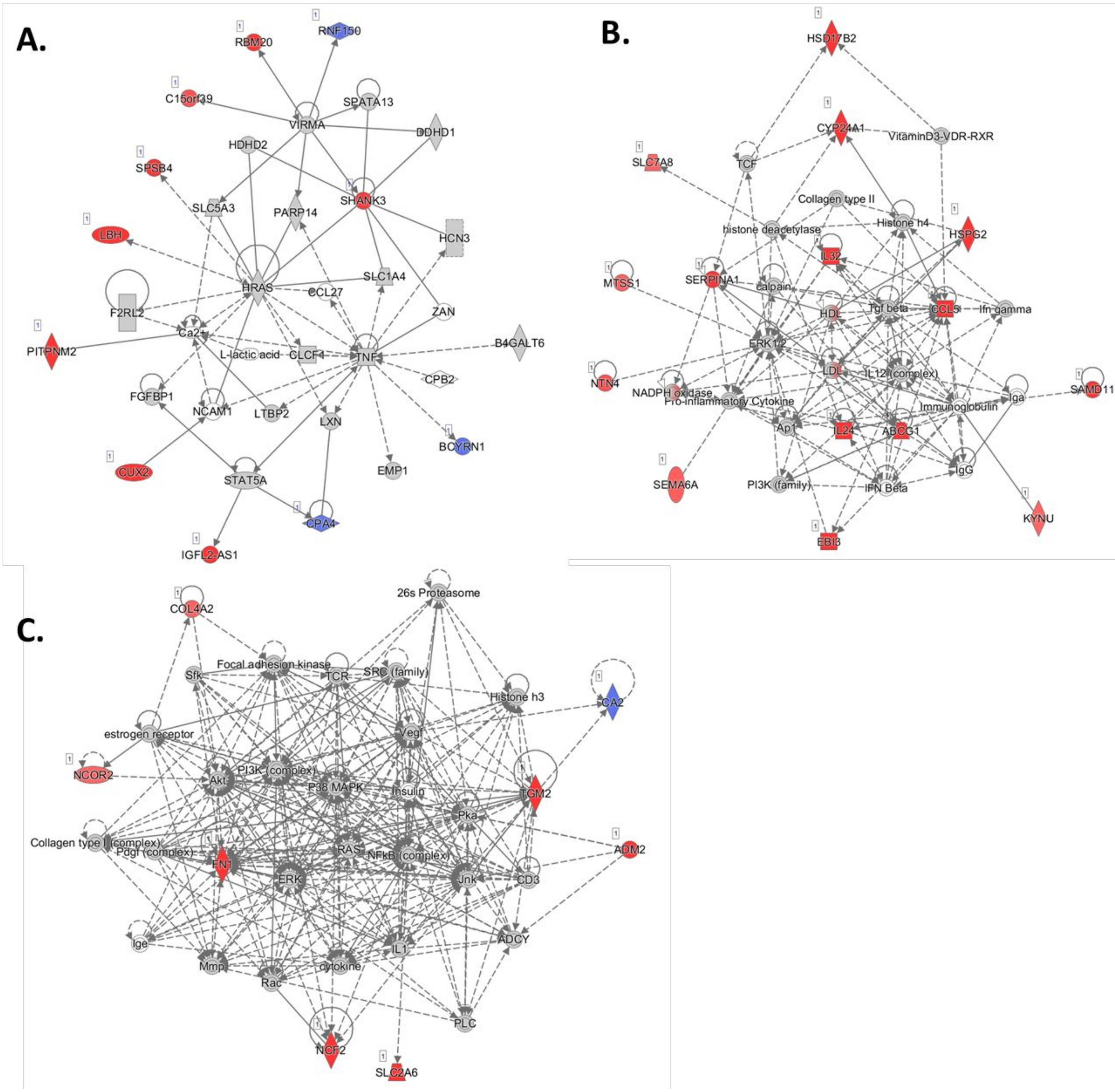

Figure S4: Her2-NLPs induce differentially expressed genes that associate with cancer. A: Her2-NLPs link with several cancer-related diseases and biofunctions. B: Eight differentially expressed genes in the Her2-NLP vs. empty-NLP comparison demonstrate significant contribution to cancer-related diseases and biofunctions. NOTCH3 and TGM2 were present in over 40% of the predicted cancer-related diseases, with NOTCH3 involved in 47% and TGM2 involved in 76% of hits, respectively. Log_2 fold change+/-1, $p<0.05$. C: Full list of cancer-related malignancies and corresponding molecular hits when comparing Her2-NLP to empty-NLP discs.

A.

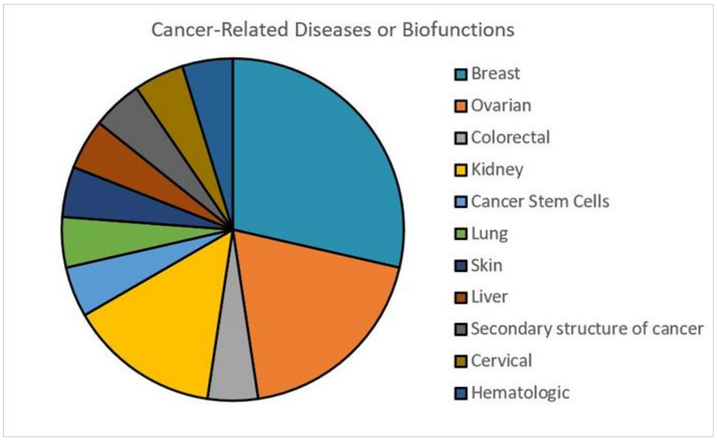

B.

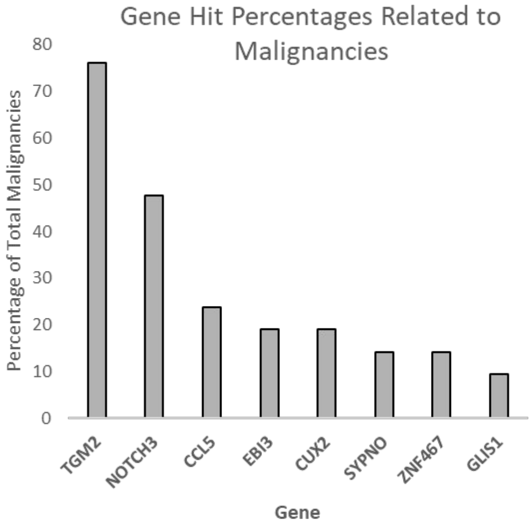

C.

Cancer Type	Related malignancies	Molecular Hits	p-value
Breast	Migration of breast cancer cell lines	CCL5,EBI3,TGM2	0.000365
	Attachment of breast cancer cell lines	TGM2	0.00211
	Dissemination of breast cancer cell lines	CCL5	0.00457
	Inflammatory breast cancer	NOTCH3	0.0143
	Cell proliferation of breast cancer cell lines	NOTCH3,TGM2	0.0279
	Primary breast cancer	NOTCH3	0.0292
Ovarian	Modification of ovarian cancer cell lines	TGM2	0.000704
	Epithelial-mesenchymal transition of ovarian cancer cell lines	TGM2	0.00141
	Cell proliferation of ovarian cancer cell lines	NOTCH3,TGM2	0.00184
	Sphere formation of ovarian cancer cell lines	TGM2	0.00211
Kidney	Colony formation of kidney cancer cell lines	TGM2	0.00281
	Apoptosis of kidney cancer cell lines	TGM2	0.0188
	Proliferation of kidney cancer cell lines	TGM2	0.0364
Cancer Stem Cells	Expansion of cancer stem cells	NOTCH3	0.00457
Liver	Hepatobiliary system cancer	CUX2,GLIS1,NOTCH3,SYNPO,TGM2,ZNF467	0.0119
Lung	Cell death of lung cancer cell lines	NOTCH3,TGM2	0.00853
Cervical	Migration of cervical cancer cell lines	TGM2	0.0353
Colorectal	Colorectal cancer	CCL5,CUX2,EBI3,NOTCH3,SYNPO,TGM2, ZNF467	0.00235
Hematologic	Hematologic cancer	CCL5,CUX2,EBI3,NOTCH3	0.0472
Secondary structure of cancer	Cancer of secretory structure	CCL5,CUX2,EBI3,GLIS1,NOTCH3,SYNPO,TGM2, ZNF467	0.0296
Skin	Migration of skin cancer cell lines	TGM2	0.00946

Table S1: Acinar Scoring Criteria.

Acinar Scoring Criteria	Polar (all)	Somewhat Polar or Apolar (Any)
Size	Compact	>20 cells
Mitotic spindles	Absent	Present
Positioning of ECM receptors	Basal	Lateral or apical
Cell-cell junctions	Well organized	Not organized
Colony Border	Smooth	Not Smooth

Table S2: Top diseases and biofunctions predicted to be activated (Z-score>2) or inhibited (Z-score<-2) with Her2-NLP treatment compared to untreated cells.

Biological Effect	p-value	Activation z-score	Molecules
Secondary tumor	2.91E-06	2.186	CCL5, COL4A2, CPA4, CUX2, EBI3, FN1, IL24, NCF2, NCOR2, NTN4, SERPINA1, TGM2
Synthesis of reactive oxygen species	9.96E-05	2.382	CCL5,FN1,IL24,IL32,NCF2,SERPINA1,TGM2
Production of reactive oxygen species	0.000156	2.189	CCL5,FN1,IL24,IL32,NCF2,TGM2
Organ Degeneration	0.000238	-2.219	ABCG1, CA2, HSD17B2, HSPG2, MTSS1, PITPNM2, SLC7A8
Proliferation of blood cells	0.000823	2.426	ABCG1, CCL5, EBI3, FN1, IL24, IL32, NCOR2, SERPINA1
Proliferation of lymphocytes	0.00144	2.23	ABCG1, CCL5, EBI3, FN1, IL24, NCOR2, SERPINA1
Formation of cellular protrusions	0.00692	2.429	CCL5, CUX2,FN1,MTSS1,NCF2,SEMA6A,SHANK3

Table S3: Upstream Factor analysis reveals potential regulators responding to Her2 treatment. Upstream regulators include mediators of inflammation such as LPS, TNF, IFNgamma, Fluticasone Propionate, tetradecanoylphorbol acetate, and/or dexamethasone.

Regulator	p-value of Her2 v. No	p-Value Her2 v. Empty	Target Molecules Her2 v. No	Target Molecules in Dataset Her2 v. Empty
**lipopolysaccharide**	5.63E-06	1.28E-05	ABCG1,CCL5,COL4A2,CUX2,EBI3,FN1,IL24,IL32,KYNU,MTSS1,NCF2,SERPINA1,TGM2	CCL5,CUX2,EBI3,NOTCH3,SYNPO,TGM2
**fluticasone propionate**	7.39E-06	2.55E-05	ABCG1,CCL5,EBI3,SERPINA1,TGM2	CCL5,EBI3,TGM2
**CCR2**	9.52E-07	7.21E-04	CCL5,COL4A2,FN1,HSPG2,TGM2	CCL5,TGM2
**NFkB (complex)**	4.03E-08	2.12E-02	ABCG1,CCL5,FN1,IL24,IL32,KYNU,MTSS1,NCF2,SLC2A6,TGM2	CCL5,TGM2
**albuterol**	2.18E-04	1.04E-05	CCL5,EBI3	CCL5,EBI3
**TNF**	2.85E-06	2.75E-03	BCYRN1,CA2,CCL5,EBI3,FN1,HSPG2,IL24,IL32,KYNU,NCF2,NCOR2,SLC7A8,TGM2	CCL5,EBI3,SYNPO,TGM2
**PS-1145**	4.08E-04	1.95E-05	CCL5,EBI3	CCL5,EBI3
**IFNG**	9.08E-06	1.05E-03	BCYRN1,CCL5,CYP24A1,EBI3,FN1,HSPG2,IL32,KYNU,MTSS1,NCF2,SERPINA1	CCL5,EBI3,GLIS1,NOTCH3
**IL10RA**	8.60E-06	5.24E-03	ABCG1,CA2,CCL5,FN1,SLC2A6,TGM2	CCL5,TGM2
**E. coli lipopolysaccharide**	2.17E-04	3.05E-04	CCL5,EBI3,IL32	CCL5,EBI3
**CD40LG**	3.90E-04	3.07E-04	CA2,CCL5,EBI3,IL24,TGM2	CCL5,EBI3,TGM2
**OSM**	2.31E-04	8.37E-04	ABCG1,CCL5,FN1,IL32,SERPINA1,SLC7A8	CCL5,NOTCH3,ZNF467
**cyanidin 3-O-glucoside**	6.76E-05	2.89E-03	ABCG1,CCL5	CCL5
**HRAS**	8.59E-06	2.33E-02	CCL5,CYP24A1,FN1,IL24,LBH,NCOR2,SPSB4,TGM2	CCL5,TGM2
**IL12A**	2.41E-03	1.17E-04	CCL5,EBI3	CCL5,EBI3
**IL1B**	1.10E-04	4.00E-03	BCYRN1,CCL5,EBI3,FN1,HSPG2,IL24,IL32,TGM2	CCL5,EBI3,TGM2
**BAX**	3.18E-03	1.56E-04	CCL5,TGM2	CCL5,TGM2
**beta-estradiol**	1.41E-05	3.63E-02	ADM2,BCYRN1,CA2,CCL5,CYP24A1,FN1,HSD17B2,IL24,KYNU,NCF2,PITPNM2,SERPINA1,SLC7A8	CCL5,NOTCH3,SYNPO
**tetradecanoylphorbol acetate**	7.55E-05	7.19E-03	CA2,CCL5,CYP24A1,EBI3,HSD17B2,IL24,IL32,LBH,SERPINA1	CCL5,EBI3,GLIS1
**eflornithine**	3.70E-05	1.47E-02	FN1,IL32,TGM2	TGM2
**PRPF4**	1.58E-03	3.61E-04	CCL5	CCL5
**TONSL**	1.58E-03	3.61E-04	CCL5	CCL5
**anthraquinone**	1.58E-03	3.61E-04	CCL5	CCL5
**STAR**	1.59E-04	4.33E-03	ABCG1,CCL5	CCL5
**VitaminD3-VDR-RXR**	5.25E-05	1.65E-02	CCL5,CYP24A1,HSD17B2	CCL5
**LDLR**	3.64E-04	2.55E-03	ABCG1,CCL5,EBI3,HSPG2	CCL5,EBI3
**PRKAA2**	2.78E-05	3.94E-02	CA2,FN1,NCF2,TGM2	TGM2
**BCL6**	4.32E-04	2.79E-03	CUX2,EBI3,IL24,LBH	CUX2,EBI3
**CAPN3**	2.52E-04	5.41E-03	CCL5,NCF2	CCL5
**N-ethyl-N-nitrosourea**	2.52E-04	5.41E-03	SERPINA1,TGM2	TGM2
**SIRT1**	4.03E-03	3.42E-04	ABCG1,CCL5,EBI3,FN1	CCL5,EBI3,SYNPO
**phenytoin**	2.87E-04	5.77E-03	CCL5,FN1	CCL5
**PELP1**	4.29E-05	4.40E-02	HSD17B2,KYNU,NCOR2,TGM2	TGM2
**Immunoglobulin**	8.17E-05	2.35E-02	CCL5,EBI3,FN1,IL24,IL32,NCF2,SAMD11	CCL5,EBI3
**dexamethasone**	4.28E-04	5.06E-03	CA2,CCL5,COL4A2,CPA4,CYP24A1,FN1,IL32,NCOR2,NTN4,SERPINA1,TGM2	CCL5,NOTCH3,TGM2,ZNF467
**ARL16**	3.16E-03	7.23E-04	CCL5	CCL5
**DCTN4**	3.16E-03	7.23E-04	CCL5	CCL5
**poly-L-glutamic acid-peptoid 1 conjugate QM56**	3.16E-03	7.23E-04	CCL5	CCL5
**JAK2**	1.80E-03	1.29E-03	CCL5,FN1,TGM2	CCL5,TGM2
**Pam3-Cys-Ser-Lys4**	1.90E-03	1.34E-03	CCL5,EBI3,IL32	CCL5,EBI3
**E. coli B5 lipopolysaccharide**	7.42E-04	3.70E-03	CCL5,IL32,NCF2,TGM2	CCL5,TGM2
**RAS**	5.81E-05	4.74E-02	CYP24A1,FN1,IL24,TGM2	TGM2
**TRAF2**	7.63E-03	3.83E-04	CCL5,EBI3	CCL5,EBI3
**SB203580**	6.15E-03	4.86E-04	CCL5,EBI3,FN1,TGM2	CCL5,EBI3,TGM2
**HDAC1**	8.89E-04	4.07E-03	CCL5,FN1,IL24,TGM2	CCL5,TGM2
**Salmonella enterica serotype abortus equi lipopolysaccharide**	2.39E-03	1.57E-03	CCL5,EBI3,IL32	CCL5,EBI3
**GNAI3**	5.01E-04	7.57E-03	CCL5,IL24	CCL5
**ADORA3**	5.01E-04	7.57E-03	CCL5,IL24	CCL5
**CTNNB1**	9.09E-05	4.30E-02	COL4A2,CYP24A1,FN1,HSD17B2,LBH,SEMA6A,SERPINA1,TGM2	NOTCH3,TGM2
